# Mitigation of saline conditions in watermelon with mycorrhiza and silicon application

**DOI:** 10.1016/j.sjbs.2021.05.019

**Published:** 2021-05-12

**Authors:** Priyanka Bijalwan, Kaouthar Jeddi, Ishan Saini, Meenakshi Sharma, Prashant Kaushik, Kamel Hessini

**Affiliations:** a Defence Institute of Bio-Energy Research, DRDO, Pithoragarh, Uttarakhand 262501, India; b Laboratory of Plant Biodiversity and Dynamic of Ecosystems in Arid Area, Faculty of Sciences of Sfax, B.P. 1171, Sfax 3000, Tunisia; c Department of Botany, Kurukshetra University, Kurukshetra, 136118 Haryana, India; d Instituto de Conservación y Mejora de la Agrodiversidad Valenciana, Universitat Politècnica de València, 46022 Valencia, Spain; e Department of Biology, College of Sciences, Taif University, P.O. Box 11099, Taif 21944, Saudi Arabia

**Keywords:** *Citrullus lanatus* L., Antioxidant, Reactive oxygen species

## Abstract

*Citrullus lanatus* L. is critical vegetable for salinity stress. Arbuscular mycorrhizal fungi (AMF) and silicon treatments are known to help as bio-ameliorator of saline soils that can improve salinity tolerance in plants. But their combined effect has never been examined on watermelon therefore, present study investigated the effect of inoculation with the *Arbuscular mycorrhizal* fungi (AMF) along with silicon on the growth and yield parameters, antioxidant enzyme activities, pigment and mineral content of *Citrullus lanatus* L. plants grown during salt stress conditions. Outcomes from the study point out that salt stressed watermelon plants showed the best morphological and biochemical values when inoculated with Silicon (4 mM) + *Glomus mosseae* + *Gigaspora gigantean*. In addition, the plants inoculated by similar treatment demonstrated less osmotic activity, electrolyte leakage, as well as peroxide content. Treatments comprising Silicon (4 mM) with either *Glomus mosseae* and *Gigaspora gigantean* also performed significantly similar for most of the traits studied in the present investigation and better than the treatment only with either one of *Glomus mosseae* and *Gigaspora gigantean*. Antioxidant efficiency of melon was certainly appreciably enhanced after incubation with AMF and Si combination in salinity stress. Overall, the application of mycorrhiza and silicon can be considered to overcome the salinity stress in watermelon.

## Highlights

• Salt stress effects agronomic traits and uptake of minerals.
• Salt stress also enhanced the oxidative stress biomarkers like hydrogen peroxide (H_2_O_2_).
• Supplementation of Mycorrhiza enhances the agronomical traits and alleviates slat stress.
• Silicon application also mitigates the salt stress through modulating antioxidant enzymes.
• The combination of Mycorrhiza and Silicon were more effective than their individual effect.

## 1 Introduction

Watermelon is an important member of family Cucurbitaceae values for its fleshy fruits in various sizes and colours (*Citrullus lanatus* L.). Over 800 million ha of worldwide soils are affected by soil salinity, representing 70% of the entire farming land. High salt accumulations in vegetation lead to ionic toxicity and osmotic pressure, therefore inhibiting older improvement by disrupting many natural processes, causing the decreasing of harvest usefulness and quality (Ahanger et al., 2018). High salt deposition in the soil results in osmotic as well as specific ion effects, which further lead to secondary oxidative stress in plants. Thus, salinity shows adverse effects on germination, growth, and reproduction of plants that subsequently diminish crop yield (Fattahi and Shamshiri, 2016, Ahmad et al., 2018a). Salinity stress is also considered as a hyperionic stress. One of the most detrimental effects of salinity stress is the accumulation of Na^+^ and Cl^−^ ions in tissues of plants exposed to soils with high NaCl concentrations. Entry of both Na^+^ and Cl^−^ into the cells causes severe ion imbalance and excess uptake might cause significant physiological disorder(s) (Kapoor and Evelin, 2014, Ahmad et al., 2018b). Nevertheless, the information about the regulatory mechanisms underlying Arbuscular mycorrhizal fungi (AMF) mediated tolerance under salinity emphasizes continuing to persist fragmentary and limited. Consequently, it is hypothesized that inoculating melon seedlings with AMF would enhance their resistance to salinity stress by increasing photosynthetic activity, avoiding damage to the chloroplast, and stimulating cellular chemical reaction. AMF promotes plant development of several plant types (Huang et al., 2011, Mo et al., 2016).

Plants endure the salinity stress by several biochemical pathways, like the development of osmotically active metabolites, together with specific proteins that control ion and fluid flux (Ahmad et al., 2008, Li et al., 2015). In this direction, inoculation with AMF greatly improved the enzyme activities as well as gene expressions enthusiastic about ROS homeostasis under abiotic stresses. These responses were correlated with growing tolerance to abiotic stress (Fan and Liu, 2011, Mo et al., 2016). High ROS levels earnestly ruin cellular membranes and affect an extensive range of crucial macro contaminants, like photosynthetic pigments, DNA, and proteins (Kohli et al., 2019). Numerous studies confirmed that AMF facilitates the salinity tolerance of vegetation by creating mutualistic associations with vegetation (Li et al., 2015, Fattahi and Shamshiri, 2016, Li et al., 2017).

AMF colonization smaller communities the melon yield along with water use usefulness under H_2_0 deficiency scenarios (Mo et al., 2016). In comparability, they have been almost all appreciably reduced under salinity stress and were the majority of the appreciably relieved after incubation of AMF. Salinity stress affects the chloroplast framework, as well as AMF, greatly alleviating these damages. Under salinity stress, the distant relative phrase quality of enzymes related to salinity tolerance was substantially high (Sabagh et al., 2020). Several studies have established the use of mineral nutrients or microbes in combating salinity stress in plants. Plants have been classified as high (Poaceae), normal (Cucurbits), and little Si (dicots) accumulators (Kaushik and Saini, 2019). The use of AMF to introduce important soil microbes has also been recognized as a safe, cost-effective, and environmentally sustainable way to improve plant resistance to a variety of abiotic stresses. The evidence that Si and AMF stimulate stress tolerance via limiting harmful ion uptake, enhancement of root hydraulic conductance, and fluid uptake, thus leading to enhanced water use efficiency, underlines the worth of regulating growth development stress (Rizwan et al. 2019). Furthermore, current studies have revealed the cumulative effects of every Si and AMF in imparting nervousness tolerance when supplemented jointly, but this effect has never been studied on watermelon (Garg et al., 2020). Therefore, the combined effect of mycorrhiza and silicon application on important morphological and biochemical traits of watermelon under saline conditions was evaluated.

## 2 Material and methods

Experiment was conducted inside the greenhouse situated at Kurukshetra University, Haryana, India. The plants were raised in a randomized complete block design with 5 replications. There were in total 9 different treatments as mentioned below (Table 1). The seeds of watermelon cultivar Sugar Baby were used in the present study. It produces small round fruits with profound black colour fruit. Generally, known for ripening between 75 and 90 days and fruit size of 2–3 kg. The test was carried inside a greenhouse house (temperature 28/20 °C as well as sixty seventy % distant relative humidity). First, seeds were germinated in medium of 2 cocopeat: 1 perlite using portrays. After that, the germinated seeds were transplanted in 2L plastic pots as one seed pot at the two-leaf stage. Medium of the pots was composed of peat-based compost and autoclaved sand in a 1:1 proportion. Plants were trained on steel wires raised over the cropping rows at the height of 1.5 and 2.9 m. the Glomus spp. and the *Gigaspora* spp. used for the inoculation of watermelon roots were isolated and multiplied based on the detailed protocol defined elsewhere (Parkash et al., 2011). After that, the pots were supplied with a 200 g air-dried soil mix and 12 g AM inoculum (for mycorrhizal plants) or similar mass of autoclaved inoculum (for non-mycorrhizal treatments). Whereas along with AMF Silicon (Si) was provided as silicic acid, at the Si concentrations used were 0 for absolute control or 2 mM for the different treatments comprising AMF. For delivering the saline conditions plants were irrigated with saline water to increase salinity to the experimental levels of 3 dS/m made of tap water with NaCl. Where, the control was left untreated with the saline water.

**Table 1:** Details of various treatments used for the characterization of *Citrullus lanatus* L.

Treatment/Code	Composition
Control 1	Crop having normal watering
Control 2	Crop given saline stress
Si (4 mM)†	Other treatments are draught with different combination of Silicon 4 mM
Gm	*Glomus mosseae*
Gg	*Gigaspora gigantea*
Si + Gm	Silicon 4 mM + *Glomus mosseae*
Si + Gg	Silicon 4 mM + *Gigaspora gigantea*
Gm + Gg	*Glomus mosseae* + *Gigaspora gigantea*
Si + Gm + Gg	Silicon 4 mM + *Glomus mosseae* + *Gigaspora gigantea*

### 2.1 *Agronomic* traits

Plant characterization was carried out after 60 days of transplanting. For the leaf area (cm) measurement using a LI-COR (LI 300) (Elron Instrument Company Pvt. Ltd., New Delhi, India) leaf area meter was used. Fruit size (L × B) cm was determined as the sum of fruit length (cm) with fruit breadth/diameter (cm). Fruit Weight (kg) was measured in grams at the time of maturity. Harvest Index was determined after harvesting of fruits as the ratio of economic yield to the biological yield.

### 2.2 Physiological traits and mineral content

Physiological parameters were examined as leaves samples were utilized to establish the total chlorophyll and carotenoid contents based on the method defined elsewhere (Ignat et al., 2013). Anthocyanin content was estimated from the floral top working with the technique provided by Tsushida and Suzuki (Tsushida and Suzuki, 1995). AM spore number and AM colonization (%) were determined based on the methods defined elsewhere (Saini et al., 2020). Phosphomolybdate blue method was used for the determination of P concentration (Murphy and Riley, 1962). Atomic absorption spectrometry was used to identify the levels of K, Mg, Fe, Cu, and Zn on Varian spectra AA 220 (Varian, Palo Alto, CA, USA).

### 2.3 Enzymatic characterization

To assess the degree of H_2_O_2_ concentration, with the help of method of Velikova et al., 2000a, Velikova et al., 2000b we used an ice bath to homogenize 0.1 g of leaf tissue with 1 mL of 0.1 percent (w/v) trichloroacetic acid (TCA). Following centrifugation, the supernatant was collected, and the absorption at 390 nm was determined. Glutathione reductase was calculated by the method of Foyer and Halliwell (1976) in a solution containing 0.025 mM sodium phosphate buffer, 0.5 mM glutathione disulfide, and 0.12 mM NADPH. The degree of lipid peroxidation in leaves was measured in terms of MDA (malondialdehyde) using Madhava Rao and Sresty's method (2000). 1.0 g of leaf samples treated with 80 percent methanol (v/v) is used to assess the quantity of abscisic acid (ABA). The samples were then centrifuged for 15 min at a speed of 10,000 g. The supernatant was then diluted to remove polar compounds before using the Sep-Pak® C18 Cartridge for column chromatography. This solution, which was collected at −20 °C, was used to measure ABA using an indirect enzyme-linked immunosorbent assay (ELISA)

(Yang et al., 2018).

To estimate the electrolyte leakage, the Dionisio-Sese and Tobita method was used (Dionisio-Sese and Tobita, 1998). Whereas the Bates and Waldren, 1973 technique was used to estimate the proline content. To evaluate enzymatic activity, 10 g of fresh leaves were homogenized in a solution comprising 100 mM Tris-HCl, 1 mM EDTA, 10 mM MgCl2, 5 mM magnesium acetate, 5 mM DTT, and 1.5 percent PVP-40. The mixture was sieved with a muslin cloth until being centrifuged for 15 min at 10,000 g.An additional enzyme assay was conducted utilizing the supernatant that had been supplemented with serine, cysteine, and ascorbate. Van-Rossum et al., 1997 approach was used to quantify superoxide dismutase (SOD). The homogenized mixture was set under 15 W fluorescent lamps for 10 min to initiate the reaction. The absorbance was measured at 560 mm using a UV–Vis spectrophotometer (Specord- 205 Analytik Jena AG, Jena, Germany). The Luck approach was used to measure the catalase (CAT) concentration, which involved mixing 50 μl of above-homogenized supernatant with 50 mM phosphate buffer and 3 mL of 20 mM H2O2 (Luck, 1974). The Nakano and Asada procedure were used to calculate ascorbate peroxidase (APX) (Nakano and Asada, 1981).

### 2.4 Data analysis

Statistical analysis was carried out using one-way analysis of variance (ANOVA) followed by post hoc test analysis computer software SPSS 16.0 (SPSS Inc. Chicago, IL, USA). Post hoc mean comparison was performed using the Duncan's Test (DMRT)*.*

## 3 Results

### 3.1 Morphological/yield parameters and mycorrhization pattern

Results showed that the combination of Silicon (4 mM) with bioinoculants, i.e., *Glomus mosseae* and *Gigaspora gigantea* performed significantly best for all the yield parameters and over both control treatments (Table 2). It was observed that leaf area (20.875 ± 2.754) was prominent in Si + Gm + Gg combination, whereas, minimum (13.215 ± 1.141) in the plants given salinity stress. Size (684.474 ± 4.485) and weight of fruit (3.545 ± 3.825) recorded best in consortium treatment but low i.e., (305.111 ± 5.42) and (0.771 ± 2.352) in control 2. Similarly, harvest index (0.954 ± 1.244) was better in Si + Gm + Gg and lowest (0.482 ± 0.718) in plants having salinity stress. Quality character, i.e., carbohydrate content (63.338 ± 1.831) was also recorded higher in similar treatment and lower (0.482 ± 0.718) in the second control treatment. The consortium treatment had been recorded highest number of spores (120.900 ± 4.333) and maximum colonization 64.300 per cent (±5.204) followed by Gm + Gg (110.500 ± 5.044) and 60.500 per cent (±5.395). The two control treatments first one in which plants had normal irrigation, second in which plants were under stress condition and the treatment having silicon (4 mM) application showed least (0.000 ± 0.000) spores and colonization per cent of arbuscular mycorrhiza (Table 2).

**Table 2:** Effect of mycorrhiza and silicon inoculation on leaf area, fruit size, fruit weight, harvest index, carbohydrate content and mycorrhization pattern of watermelon plant under saline conditions.

Treatments	Leaf area (cm)	Fruit size (L × B) cm	Fruit Weight (kg)	Harvest Index	Carbohydrate (mg mg-100 Fresh Weight)	AM spore number	AM colonization (%)
Control 1	15.121 ± 1.214^f^‡	313.122 ± 6.145 ^h^	0.888 ± 2.445 ^h^	0.534 ± 0.898^f^	46.762 ± 1.196 ^g^	0.000 ± 0.000 ^h^	0.000 ± 0.000 ^h^
Control 2	13.215 ± 1.141^i^	305.111 ± 5.424^i^	0.771 ± 2.352^i^	0.482 ± 0.718 ^g^	38.613 ± 1.196 ^h^	0.000 ± 0.000 ^h^	0.000 ± 0.000 ^h^
Si (4 mM)†	16.624 ± 2.442^e^	411.223 ± 4.324 ^g^	1.986 ± 3.244 ^g^	0.587 ± 0.986^e^	55.412 ± 2.402^f^	0.000 ± 0.000 ^h^	0.000 ± 0.000 ^h^
Gm	17.756 ± 2.134^d^	477.352 ± 6.643^f^	2.457 ± 4.534^e^	0.689 ± 1.686^d^	56.077 ± 1.256^e^	79.800 ± 6.244^f^	48.300 ± 6.565^f^
Gg	18.878 ± 3.326^c^	482.245 ± 5.354^e^	2.098 ± 4.423^f^	0.612 ± 1.394^de^	58.021 ± 1.228^d^	88.900 ± 3.855^e^	50.400 ± 3.914^e^
Si + Gm	20.129 ± 2.183^ab^	562.433 ± 6.166^c^	3.009 ± 2.626^c^	0.823 ± 1.563^bc^	61.512 ± 3.005^bc^	94.400 ± 4.444^d^	55.400 ± 4.553^d^
Si + Gg	20.521 ± 3.235^ab^	625.351 ± 5.145^b^	3.134 ± 3.415^b^	0.897 ± 1.445^b^	62.545 ± 1.778^ab^	105.400 ± 6.222^c^	58.800 ± 6.276^c^
Gm + Gg	19.623 ± 2.034^b^	530.607 ± 5.453^d^	2.889 ± 4.523^d^	0.777 ± 1.366^c^	60.044 ± 1.381^c^	110.500 ± 5.044^b^	60.500 ± 5.395^b^
Si + Gm + Gg	20.875 ± 2.754^a^	684.474 ± 4.485^a^	3.545 ± 3.825^a^	0.954 ± 1.244^a^	63.338 ± 1.831^a^	120.900 ± 4.333^a^	64.300 ± 5.204^a^
LSD (P ≤ 0.05)	7.805	310.071	0.138	0.915	31.321	27.226	21.606
ANOVA (3, 16)	3.329	2.911	11.179	4.627	15.061	160.872	233.257

Control1 – crop having normal watering; Control2 – crop given saline stress; †Other treatments are draught with different combination of Silicon 4 mM and Bioinoculants – *Glomus mosseae* + *Gigaspora gigantea*±: Standard deviation ‡ column brackets preceded by the same letter are not substantially different at *P* ≤ 0.05 (Duncan’s Multiple Range Test).

### 3.2 Pigmentation and mineral content

The outcomes pertaining to pigment and mineral content is represented in Table 3. The plants inoculated with Silicon (4 mM) + *Glomus mosseae* + *Gigaspora gigantean* combination proved to be significantly efficient for all quality characters. This treatment had recorded higher (23.565 ± 1.653) chlorophyll content, whereas lower (23.565 ± 1.653) in plants given stress. Similarly, carotenoids content had maximum (18.998 ± 0.983) value in consortium treatment followed by Si + Gg treatment and minimum (9.203 ± 0.809) in second control treatment (Table 3). There were significant differences observed among the 9 treatments for the mineral content. Potassium content had higher (19.034 ± 0.788) values in the plants which were inoculated in Si + Gm + Gg combination, while lower (13.231 ± 1.675) values in salinity stressed plants (Table 3). Similarly, consortium treatment showed excellent (16.034 ± 1.223) magnesium content, meanwhile control 2 had lowest (8.778 ± 1.387) value (Table 3). Greater amount of phosphorus (5.355 ± 2.098) and iron content (39.569 ± 1.432) was calculated in the plants which were treated with Si + Gm + Gg combination and plants which had given stressed conditions showed lower P and Fe content (Table 3). The zine content (31.087 ± 1.595) found maximum in consortium treatment. Similar treatment also had higher values for copper (8.904 ± 1.457), whereas, minimum zinc and copper was calculated in salinity stressed plants, i.e., (26.156 ± 0.689) and (3.001 ± 1.941), respectively (Table 3).

**Table 3:** Effect of mycorrhiza and silicon inoculation on pigment and mineral content of watermelon plant under saline conditions Pigment and the mineral content variation in the 9 treatments of watermelon under saline conditions.

Treatments	Total chlorophyll (mg g^−1^ Fresh Weight)	Total Carotenoid (mg g^−1^ Fresh Weight)	Potassium mg g^−1^ Fresh weight	Magnesium mg g^−1^ Fresh weight	Phosphorus mg g^−1^ Fresh weight	Iron μg g^−1^ Fresh weight	Zinc μg g^−1^ Fresh weight	Copper μg g^−1^ Fresh weight
Control 1	17.781 ± 2.127 g‡	11.221 ± 0.816 ^h^	14.708 ± 0.716^e^	10.611 ± 0.943^f^	2.074 ± 0.921^d^	32.666 ± 0.882^f^	28.044 ± 0.911^d^	4.205 ± 0.869^e^
Control 2	14.581 ± 1.954 ^h^	9.203 ± 0.809^i^	13.231 ± 1.675^f^	8.778 ± 1.387 ^g^	1.887 ± 2.065^e^	30.898 ± 0.961 ^g^	26.156 ± 0.689^e^	3.001 ± 1.941^f^
Si (4 mM)†	18.975 ± 1.743^f^	12.333 ± 0.809 ^fg^	14.988 ± 1.568^de^	10.999 ± 1.366^ef^	2.134 ± 1.588^d^	33.005 ± 2.008^f^	28.787 ± 2.091^d^	4.677 ± 1.334^e^
Gm	19.066 ± 1.897^e^	13.568 ± 0.809^e^	16.434 ± 1.684^d^	12.764 ± 1.558^d^	3.658 ± 1.674^bc^	34.568 ± 1.988^e^	29.412 ± 0.913^c^	5.657 ± 1.378^d^
Gg	20.122 ± 2.067^d^	14.799 ± 0.792^de^	17.885 ± 1.724^c^	13.974 ± 1.416^c^	3.891 ± 1.544^b^	35.432 ± 1.687^d^	29.877 ± 0.986^c^	6.768 ± 1.834^c^
Si + Gm	21.216 ± 2.003^c^	17.653 ± 0.782^bc^	18.576 ± 1.903^a^	15.347 ± 1.627^bc^	4.786 ± 2.423^ab^	37.265 ± 1.726^b^	30.498 ± 1.466^b^	7.879 ± 1.711^b^
Si + Gg	22.786 ± 1.565^b^	18.382 ± 0.681^a^	18.789 ± 2.051^a^	15.787 ± 1.092^b^	5.178 ± 2.184^a^	38.061 ± 1.342^b^	30.784 ± 1.667^b^	8.098 ± 1.467^a^
Gm + Gg	21.986 ± 1.756 ^cd^	15.869 ± 0.812^c^	18.003 ± 1.983^ab^	15.112 ± 1.762^bc^	4.076 ± 2.377^ab^	36.667 ± 1.565^c^	30.177 ± 1.711^b^	8.554 ± 1.517^a^
Si + Gm + Gg	23.565 ± 1.653^a^	18.998 ± 0.983^a^	19.034 ± 0.788^a^	16.034 ± 1.223^a^	5.355 ± 2.098^a^	39.569 ± 1.432^a^	31.087 ± 1.595^a^	8.904 ± 1.457^a^
LSD (P ≤ 0.05)	3.122	1.314	0.185	0.218	1.811	0.129	1.721	1.401
ANOVA (3, 16)	23.311	80.073	2.093	6.642	7.424	3.416	2.886	2.476

Control1 – crop having normal watering; Control2 – crop given saline stress; †Other treatments are draught with different combination of Silicon 4 mM and Bioinoculants – *Glomus mosseae* + *Gigaspora gigantea*±: Standard deviation‡ column brackets preceded by the same letter are not substantially different.

### 3.3 Enzymatic activity

According to Table 4, the activity of H_2_O_2_ increased during the stress condition. However, our bio-inoculated treated plant manages to withstand saline stress. Lowest peroxide content was found in plants treated with Silicon (4 mM) + *Glomus mosseae* + *Gigaspora gigantean* (92.169 ± 0.932) (Table 4). Likewise, the ABA level measured was also found lowest in the same combination of Silicon (4 mM) + *Glomus mosseae* + *Gigaspora gigantean* (0.427 ± 1.027). The enzyme lipid peroxidase and Glutathione reductase were found maximum in the consortium treatment of Silicon (4 mM) + *Glomus mosseae* + *Gigaspora gigantean*, 58.321 ± 1.796 and 8.943 ± 1.114, respectively, as compared to other treatments (Table 4).

**Table 4:** Effect of mycorrhiza and silicon inoculation on accumulation of H_2_O_2_, Lipid Peroxidase, ABA and Glutathionate reductase in watermelon plant under saline conditions.

Treatments	H_2_O_2_ accumulation (μg mol^−1^)	Lipid Peroxidase (MDA mol g^−1^ Fresh Weight)	Abscisic acid content (mg g^−1^ Fresh Weight)	Glutathionate reductase (unit mg^−1^ Protein)
Control 1	135.421 ± 2.791 ^h^‡	57.718 ± 2.712^f^	0.741 ± 0.912 ^g^	105.455 ± 1.226^e^
Control 2	155.432 ± 1.848 ^g^	58.822 ± 2.926 ^g^	0.803 ± 0.825 ^h^	104.553 ± 1.462^f^
Si (4 mM)†	120.821 ± 2.378^f^	55.674 ± 1.241^de^	0.621 ± 2.006^f^	106.043 ± 2.666^de^
Gm	116.742 ± 1.656^ef^	56.434 ± 2.802^e^	0.612 ± 1.485^e^	106.342 ± 3.153^d^
Gg	114.651 ± 2.303^e^	53.657 ± 0.983^d^	0.608 ± 1.164^d^	107.453 ± 0.491^bc^
Si + Gm	96.201 ± 1.267^bc^	50.666 ± 1.826^b^	0.533 ± 1.436^bc^	107.576 ± 0.865^bc^
Si + Gg	94.214 ± 1.457^ab^	49.382 ± 3.029^bc^	0.486 ± 1.507^b^	108.627 ± 1.139^ab^
Gm + Gg	110.099 ± 1.235^d^	51.788 ± 3.503^c^	0.579 ± 1.351^c^	107.885 ± 2.632^b^
Si + Gm + Gg	92.169 ± 0.932^a^	58.321 ± 1.796^a^	0.427 ± 1.027^a^	108.943 ± 1.114^a^
LSD (P ≤ 0.05)	121.205	0.285	0.614	101.115
ANOVA (3, 16)	17.315	8.163	4.717	25.506

Control1 – crop having normal watering; Control2 – crop given saline stress; †Other treatments are draught with different combination of Silicon 4 mM and Bioinoculants – *Glomus mosseae* + *Gigaspora gigantea*±: Standard deviation‡ column brackets preceded by the same letter are not substantially different at *P* ≤ 0.05 (Duncan’s Multiple Range Test). MDA- Malondialdehyde.

Results from Table 5 showed that watermelon plants treated with Silicon (4 mM) + *Glomus mosseae* + *Gigaspora gigantean* were the most effective activators of the protective proteins. The decreased osmotic potential (0.179 ± 1.432), electrolyte leakage (37.321 ± 0.916) and peroxide content (23.543 ± 1.164) was observed in watermelon plants under Silicon and both bioinoculant treatment, whereas, salt stressed plants had increased osmotic potential (0.542 ± 0.884), electrolyte leakage (47.082 ± 2.246) and peroxide content (37.753 ± 1.6423 Maximum (8.242 ± 1.253) peroxidase protein production was calculated in Si + Gm + Gg treatment (Table 5). This treatment also performed best for more catalase (147.474 ± 1.268) production (Table 5). Control 2 produced a lower amount of peroxidase and catalase i.e., (4.063 ± 0.963) and (125.672 ± 0.911), respectively. Plants with Silicon and both bioinoculants were observed with an improved amount of ascorbate peroxidase (0.568 ± 1.173) and superoxide dismutase (142.432 ± 2.323) (Table 5). The least reading i.e., (0.284 ± 0.783) and (108.578 ± 3.0197) of these proteins were recorded in stress given plants of watermelon (Table 4). These results showed a great impact of consortium on watermelon enzymatic activity during stress conditions. Analysis of variance showed a significant difference between plants subjected or not to salinity stress. Electrolyte leakage and the peroxidase content was higher in the controls. Among the 9 treatments the highest content of proline, CAT and SOD were determined in the consortium treatment (Table 5).

**Table 5:** Effect of mycorrhiza and silicon inoculation on osmatic potential, electrolyte leakage, peroxide, proline, peroxidase, calatase, ascorbate peroxidase and superoxide dismutase content in watermelon plant under saline conditions.

Treatments	Osmotic potential (MPa)	Electrolyte leakage (%)	Peroxide content (μmol g^−1^ Fresh Weight)	Proline (μmol g^−1^ Fresh Weight)	Peroxidase (U mg^−1^ protein)	Catalase (U mg^−1^ protein)	Ascorbate peroxidase (mg protein min-10)	Superoxide dismutase (U mg-1 protein)
Control 1	0.442 ± 0.971^f^‡	46.078 ± 2.126^f^	35.675 ± 1.216^f^	110.041 ± 0.912 ^g^	5.943 ± 0.831 ^g^	136.542 ± 0.701 ^g^	0.312 ± 0.803^f^	121.456 ± 1.507^f^
Control 2	0.542 ± 0.884 ^g^	47.082 ± 2.246 ^g^	37.753 ± 1.642 ^g^	102.403 ± 0.825 ^h^	4.063 ± 0.963 ^h^	125.672 ± 0.911 ^h^	0.284 ± 0.783 ^g^	108.578 ± 3.0197 ^g^
Si (4 mM)†	0.381 ± 2.387^e^	44.667 ± 1.321^de^	31.123 ± 2.666^de^	111.221 ± 2.006^f^	6.346 ± 2.604^f^	139.221 ± 2.714^f^	0.395 ± 2.115^e^	124.273 ± 2.106^e^
Gm	0.372 ± 1.566^de^	45.543 ± 2.032^e^	32.342 ± 3.153^e^	124.412 ± 1.485^e^	6.673 ± 1.734^e^	142.412 ± 1.367^e^	0.429 ± 1.374^de^	129.984 ± 1.734d^e^
Gg	0.361 ± 1.333^d^	42.667 ± 0.832^d^	30.453 ± 0.921^d^	126.631 ± 1.164^d^	7.032 ± 1.419^d^	143.631 ± 1.243^d^	0.461 ± 1.253^d^	131.078 ± 1.883^d^
Si + Gm	0.231 ± 1.726^bc^	39.866 ± 1.216^b^	27.876 ± 0.896^b^	133.313 ± 1.436^bc^	7.556 ± 1.564^bc^	145.123 ± 1.454^c^	0.523 ± 1.462^bc^	136.689 ± 2.432^bc^
Si + Gg	0.214 ± 1.574^b^	38.438 ± 3.209^bc^	26.627 ± 1.119^bc^	135.086 ± 1.507^b^	7.898 ± 1.315^b^	146.086 ± 1.506^b^	0.544 ± 1.617^b^	139.536 ± 2.023^b^
Gm + Gg	0.299 ± 1.253^c^	40.878 ± 3.003^c^	28.985 ± 2.673^c^	132.123 ± 1.351^c^	7.384 ± 1.343^c^	144.313 ± 1.435^c^	0.499 ± 1.325 ^cd^	132.424 ± 1.982 ^cd^
Si + Gm + Gg	0.179 ± 1.432^a^	37.321 ± 0.916^a^	23.543 ± 1.164^a^	137.327 ± 1.027^a^	8.242 ± 1.253^a^	147.474 ± 1.268^a^	0.568 ± 1.173^a^	142.432 ± 2.323^a^
LSD (P ≤ 0.05)	1.231	0.185	1.323	0.312	0.519	10.216	0.216	0.567
ANOVA (3, 16)	7.235	18.136	35.039	4.174	17.767	50.872	3.206	18.136

Control1 – crop having normal watering; Control2 – crop given saline stress; †Other treatments are draught with different combination of Silicon 4 mM and Bioinoculants – *Glomus mosseae* + *Gigaspora gigantea*±: Standard deviation‡ column brackets preceded by the same letter are not substantially different at *P* ≤ 0.05 (Duncan’s Multiple Range Test).

## 4 Discussions

Excessive salt concentrations damages plants that cause great loss in crop production, productivity and quality. During salt stress conditions, plant physiology is often affected in the following manner: (a) excessive amounts of ions, such as Na^+^, cause the destruction of enzyme structures and cell organelles, thereby disrupting photosynthesis, respiration, and protein synthesis in plants (Kaur et al., 2018, Ahmad et al., 2017); (b) accumulation of salts in the soil induces physiological drought and nutrient imbalance in plants (Kaya et al. (2020); and (c) salinity stress causes the plants to produce reactive oxygen species (ROS) (Ahmad et al., 2017, Ahmad et al., 2018a). In such conditions AMF is a very cost-effective method (Abdel-Fattah, 2012, Porcel et al., 2015). Plant biostimulants (PBs) have different compounds, substances, and growth-promoting microorganism formulations used to enhance ecological, physiological, and biochemical processes in plants (González-González et al., 2020). The plants inoculated with mycorrhiza have shown improved tolerance and resistance to a broad range of environmental stressors caused by both abiotic and biotic factors due to their protection from changes in their physiology (Song et al., 2015, Chen et al., 2018, Tavarini et al., 2018, Begum et al., 2019).

The root biomass which is regulated by the application of AMF that enhances nutrient uptake and translocation, which results in an increase in total carbohydrate, phenolic level and protein content while promoting growth, biomass production, stress tolerance (Colla et al., 2015, Rouphael et al., 2015, Fiorentino et al., 2018, Rouphael and Colla, 2018, Lucini et al., 2019). In this direction, proline is in a position to reinforce the subcellular machinery. Besides, scavenge MDA, and free radicals may be the decomposition item of polyunsaturated fatty acids of membranes also it's used to check out the severity of oxidative damage ( Li et al., 2015, Cao et al., 2018). Our outcomes proposed that vegetation inoculated with AMF and cultivated under salinity condition possessed a reduced MDA concentration and a better proline focus. Enzymatic antioxidants, like Sod, CAT, APX, GR, MDHAR, and DHAR, can effectively relieve the additional ROS, maintain the balance of the improvement, and remove ROS (Miller et al., 2010, Mo et al., 2016).

The colonization and spore number of AMF are determined by AMF and plant species. Similarly, here the percentages of AMF colonization varied, and the results were similar to a previous maize study (Estrada et al., 2013). Salinity in soil reduces plant development and biomass accumulation by impairing photosynthetic ability (Pitman and Läuchli, 2002). AMF has previously been shown to increase the photosynthetic capacity of plants by rising gaseous exchange capability and the content of chlorophyll activity in leaves (Ruiz-Lozano et al., 2012). Under salt stress conditions, the chlorophyll content of AMF treatments was marginally higher, which may be related to AMF strains' potential to increase photosynthetic pigment content, as shown in past findings (Talaat and Shawky, 2014, Zai et al., 2012). Numerous tests have been performed to examine the impact of salinity on chlorophyll pigments (Khan et al., 2014, Yang et al., 2015).

Fungal hyphae grow into the roots of plants due to a symbiotic partnership between AMF and plants (Gutjahr et al., 2009, Gutjahr and Paszkowski, 2013). As a result, the plant makes photosynthetic products that AMF uses to supply soil nutrients to the plant root system like phosphorus, nitrogen, copper, and zinc (Ferrol et al., 2019). This symbiotic relationship has been found to facilitate secondary metabolite production, such as that of phenolic acids or flavonoids, which is needed for abiotic stress tolerance (Tavarini et al., 2018). High salinity inhibits the absorption of essential macro and micronutrients.

Silicon (Si) is the second most abundant element in soil. Although Si has not been classified as an essential element, it has been proven to enhance the quantitative and qualitative traits of plants, especially under environmental stresses, such as salinity, drought, and heavy metal toxicity (Ahmad et al., 2019a, Ahmad et al., 2019b). Moreover, Si can be regarded as “multitalented” element and could ameliorate soil conditions and nutrient contents (e.g., N, P, and K) in plants, making it a high-quality fertilizer for promoting ecologically sound agricultural practices (Etesami and Jeong, 2018, Ali et al., 2019). Silicon (Si), as a macroelement, has a vital role in plants cycles (Rios et al., 2017). This element is the eighth most common element in nature and the second most common element found in soil after oxygen. One of the main functions of Si is improving the plants growth and yield especially in stress condition (Etesami, 2018). To achieve plant tolerance, Si promotes plant photosynthesis by favourably exposing leaves to light (Alzahrani et al., 2018).

Some studies have shown that Si participates in regulating the accumulation of osmoregulatory substances in plants. In cucumber, Na + Na_2_SiO_3_·9H_2_O treatment could increase the accumulation of soluble sugars (mainly sucrose and glucose) and decrease the osmotic potential of xylem sap in the root system compared with Na treatment, thus contributing to the promotion of root water uptake (Zhu et al., 2016). In present study it was observed *C*. *lanatus* plants colonized with AMF showed a great decline in the accumulation of reactive oxygen species and prevented oxidative damage to cellular structures and their functional integrity (Ahmad et al., 2010, Velarde-Buendía et al., 2012). Ruiz-Lozano et al., 2012, He et al., 2017 observed that *Oryza sativa*, *Lycium barbarum* and *Robinia pseudoacacia*, respectively, exposed to high salinity showed membrane leakage which results in damage to plant plasma membranes; however, AMF colonization results in greater maintenance of water balance in tissues due which protect their membrane structures. The stress-induced reduction in the membranes' structural and functional stability mainly results from the rapid increase in the lipid peroxidation (Nath et al., 2016, Ahmad et al., 2017, Ahmad et al., 2018a). ROS can severely disrupt the normal metabolism and cellular functions of plants through its oxidative damage to lipids, nucleic acids, oxidizing proteins, and photosynthetic pigments (Kapoor and Singh, 2017, Ahmad et al., 2019a, Ahmad et al., 2019b). ROS scavengers like superoxide dismutase (SOD), catalase (CAT), ascorbate peroxidase (APX), peroxidase (POD), glutathione reductase (GR), proline and ascorbic acid are the most important osmoprotectants that prevent cellular oxidative damage during salt stress (Mansour and Ali 2017). Several studies concluded that AMF symbiosis can escalate antioxidative defence systems of plant through higher antioxidant activity in AM fungi inoculated plants, accelerating dismutation of superoxide to H_2_O_2_ and subsequently impeding H_2_O_2_ buildup by higher activities of CAT, POX and APX (Fouad et al., 2014, Begum et al., 2019). Si could improve ROS scavenging ability by regulating the activities/contents of enzymatic/nonenzymatic antioxidants in plants, and the regulatory effect is different depending on plant species. For example, in barley, Si could increase the activity of CAT, SOD, and GR (Abbas et al., 2015, Alzahrani et al., 2018). For any distant relative term levels of antioxidant outcome linked genes Sod, GR, APX, CAT, their distant relative term amounts were significantly made better and in addition was substantially more appreciably enhanced after AMF inoculation and with the application of silicon. Our evaluation demonstrated the favorable results of AMF and silicon under salinity stress, which may be implicated in managing watermelon developing under salinity.

## 5 Conclusion

Salinity is significant abiotic stress that negatively influences plant development, resulting in an excessive decrease in quality and quantity of watermelon characters. The watermelon is inoculated with the Arbuscular mycorrhizal fungi (Silicon and AMF) to assess its effect on watermelon morphological and biochemical traits and the enzymatic activity. Arbuscular mycorrhizal (AM) fungi are among the most crucial categories of plant symbionts and positively influence numerous facets of plant life, stress tolerance, better growth, improved nutrition, i.e., and disease resistance. Supplementation of silicon improved yield and physiology of crop plants. Silicon mediated stress mitigation calls for several regulatory mechanisms such as photosynthesis, detoxification of damaging reactive oxygen species applying antioxidant, and the right nutrient management. Antioxidant performance of watermelon was indeed appreciably upgraded after incubation with Si and AMF conjunction in salinity stress.

## 6 Declarations

**Ethics approval:** Not Applicable.

**Consent to participate:** All authors consent to participate in this manuscript.

**Consent for publication:** All authors consent to publish this manuscript in Saudi Journal of Biological Science.

**Availability of data and material:** Data will be available on request to corresponding or first author.

**Code availability:** Not Applicable.

## Author contributions

PB and IS drafted the experimental design and performed the experiments. MS and KJ helped in data collection and data analysis. PK and HK wrote initial draft of manuscript and revised the manuscript to present form. All authors read the manuscript before communication.

## Declaration of Competing Interest

The authors declare that they have no known competing financial interests or personal relationships that could have appeared to influence the work reported in this paper.
